# Non-Uniform Antenna Array for Enhanced Medical Microwave Imaging

**DOI:** 10.3390/s25103174

**Published:** 2025-05-17

**Authors:** Younis M. Abbosh, Kamel Sultan, Lei Guo, Amin Abbosh

**Affiliations:** 1 College of Electronics Engineering, Ninevah University, Mosul 41002, Iraq; younis.abbosh@uoninevah.edu.iq; 2 School of EECS, The University of Queensland, St. Lucia, QLD 4072, Australia; l.guo3@uq.edu.au (L.G.); a.abbosh@uq.edu.au (A.A.)

**Keywords:** medical microwave imaging, electromagnetic imaging, non-uniform antenna array, confocal imaging

## Abstract

A non-uniform antenna array is proposed to enhance the accuracy of medical microwave imaging systems by increasing the amount of useful information captured about the imaged domain without increasing the number of antennas. These systems have so far been using uniform antenna arrays, which lead to highly correlated signals, limiting the amount of imaging information and adversely affecting diagnostic accuracy. In the proposed non-uniform antenna array method, the optimal number and positions of antennas are calculated with the aim of enhancing spatial diversity and reducing information redundancy. The mutual information coefficient is used as a metric to evaluate and minimize redundancy between received signals. A microwave head imaging system is used to verify the proposed approach. The results of the investigated scenarios show that using a non-uniform antenna configuration outperforms a uniform setup in imaging accuracy and clarity, when using the same number of antennas. Moreover, the reconstructed images demonstrate that using an optimized non-uniform antenna array with fewer elements can outperform a uniform array with more elements in terms of localization accuracy and image quality. The proposed approach improves imaging performance and reduces system complexity, cost, and power consumption, making it a practical solution for real-world biomedical imaging applications.

## 1. Introduction

Medical microwave imaging (MMI) has evolved as an innovative modality that uses signals at the microwave frequency band to create images of the internal structures of the human body [1,2,3]. Due to the lossy nature of human tissues, MMI usually uses the low microwave frequency band, which results in a trade-off between resolution and required penetration [4]. Unlike traditional medical imaging methods such as X-ray, MRI, and CT scans, which rely on ionizing radiation or strong magnetic fields, MMI uses non-ionizing, low-level microwave signals. This makes MMI a safer option for frequent monitoring. Additionally, MMI has the potential to provide high contrast in soft tissues, which is useful for detecting many types of abnormalities [5,6]. The ability to be portable and offer real-time imaging at a low cost further sets MMI apart from conventional imaging techniques [7,8].

**Figure 1:**
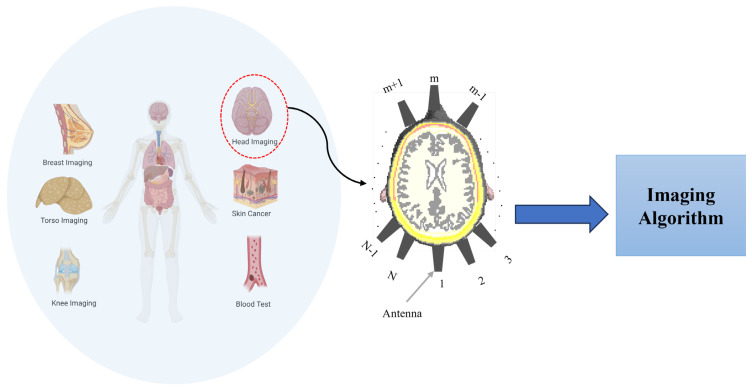
Different imaging targets in medical microwave imaging with a focus on head imaging as an example.

MMI systems operate by irradiating the domain under test (e.g., head [9], knee [10], breast [11], torso [12], and so on) using electromagnetic (EM) waves at a specific frequency band depending on the application. The scattered signals from the domain, which carry information about the dielectric properties of tissues, see Figure 1, are captured and processed to create an image of the internal structure of the domain. Since properties of the scattered signals vary with tissue pathology, MMI can differentiate between healthy and abnormal regions. The antenna array and imaging algorithms are the two main components that determine the system’s performance (see Figure 1).

Typically, increasing the number of antennas in microwave imaging systems improves imaging resolution and contrast by collecting more scattered information. However, in medical applications, the size of the imaging region is restricted, and thus, the maximum number of imaging antennas is limited. Additionally, human tissues exhibit a heterogeneous and irregular distribution, meaning that some antennas may receive redundant or highly correlated information rather than new diagnostic insights. Increasing the number of antennas beyond a certain point introduces several challenges, including higher mutual coupling, increased system complexity, and diminishing returns in received information [6]. Furthermore, inaccurate antenna placement can lead to information loss and degrade imaging quality. This issue arises because certain antennas may predominantly receive cluttered signals rather than useful diagnostic information. The optimal placement of antennas plays a crucial role in ensuring the collection of independent and complementary data, ultimately enhancing the accuracy of the reconstructed images.

To address these challenges, various antenna design strategies have been explored. Miniaturization techniques, such as metasurface-based [13], multi-resonators [14], and high-dielectric-loaded antennas [8,15], have been proposed to reduce antenna size and increase element density. However, these methods often compromise bandwidth and efficiency, limiting imaging performance. Reconfigurable antenna arrays enable adaptive beamforming and clutter suppression but may suffer from a lower signal-to-noise ratio, which is problematic in MMI where the target response is almost embedded in clutter, and introduce higher control complexity [16]. Multi-polarization and multi-frequency arrays enhance contrast and tissue differentiation but require precise calibration, increasing system complexity [6]. While these methods attempt to improve imaging by increasing the number of elements, they still rely on uniform antenna array distributions, which may result in a loss of critical diagnostic information due to redundant or correlated data collection.

Although these methods attempt to improve imaging by increasing the number or adaptability of antennas, they typically rely on uniform array configurations, where elements are evenly spaced. Such configurations are prevalent due to their ease of design and symmetry, but they do not account for the anatomical irregularity of biological tissues, often resulting in redundant data acquisition and limited imaging improvements [17,18]. Some prior works have explored sparse array optimization and compressive sensing-based configurations to reduce system complexity while maintaining image quality [19,20]. In [21], Zamani et al. employed spatial interpolation to extend a virtual antenna array, enhancing resolution without adding hardware. However, this method depends on interpolation accuracy and may be sensitive to anatomical complexity. More information-theoretic frameworks, such as mutual information and correlation analysis, have been explored in biomedical applications setups [21], but these always assume uniform antenna placement strategies tailored for complex anatomical models.

On the other hand, the accuracy of MMI also depends on the imaging algorithm used to process the captured scattered signals [8,22]. One of the most well-known methods is the confocal imaging algorithm [23,24,25,26]. This algorithm enhances the resolution and contrast of the images by aligning the signals received from different antennas to a common focal point. The accuracy of this method depends on the bandwidth used and the number of antennas. Essentially, the detection accuracy is based on the amount of useful information collected by the antenna array. This is why multistatic confocal imaging, which uses multiple antennas, generally produces better images than monostatic confocal imaging. As explained earlier, increasing the number of antennas is not a practical solution. A large number of closely spaced antennas suffer from a strong mutual coupling and highly correlated captured signals, and thus, introduce artefacts and reduce the quality of reconstructed images.

This paper aims to explore this fact and suggests using a non-uniform antenna array in MMI. In this case, the distances between antenna elements are varied to maximize the information received. To achieve this goal, an optimization algorithm is used to minimize the information correlation coefficient between neighboring antennas and select the optimum distances and the optimum number of array elements. This ensures that the antenna elements are positioned in such a way that the similarity of the received information from neighboring antennas is minimized, while the total information received by the array is maximized. This approach improves the overall performance of the MMI system, leading to more accurate and detailed images.

## 2. Problem Formulation

In this section, the principle of total acquired information in medical microwave imaging is introduced, and the antenna design used is explained. In addition, this section investigates the spatial influence of imaging antenna positions using the mutual information coefficient (MIC) to understand how antenna placement affects the diversity and redundancy of the acquired microwave signals. Specifically, two complementary analyses are performed: (1) evaluating how signal redundancy changes as the antenna moves while the other antennas remain fixed and (2) assessing the independence of information captured by the antenna across consecutive positions.

### 2.1. Principle of Acquired Imaging Information in MWI

In MMI, the signals received are significantly influenced by the spatial configuration of the antenna array. A non-uniform imaging antenna array strategically varies the spacing between antennas to enhance the collected information. This approach minimizes signal redundancy, reduces mutual coupling, and improves image reconstruction accuracy. The key theoretical principle underlying this optimization is adjusting element spacing to maximize the independent information of acquired signals by using the optimum number of elements. By optimizing antenna positions, the system enhances both image contrast and spatial resolution, leading to improved abnormality detection.

The estimation of the optimal number of antennas can be approached using two primary strategies: the degrees of freedom (DoF) analysis [19,27] and the information correlation coefficient (ICC)/mutual information coefficient (MIC) method [21]. The DoF method is typically derived under the assumption of ideal point sources operating at a single frequency, which makes it less suitable for practical systems employing wideband antennas with finite physical dimensions. Conversely, the ICC/MIC approach provides a more practical framework by evaluating the redundancy in the data collected by adjacent antennas across the full operating frequency range. It does so by quantifying the level of similarity or dependence between their captured signals, offering insight into spatial diversity and aiding in the strategic placement of antennas for improved imaging performance.

**Figure 2:**
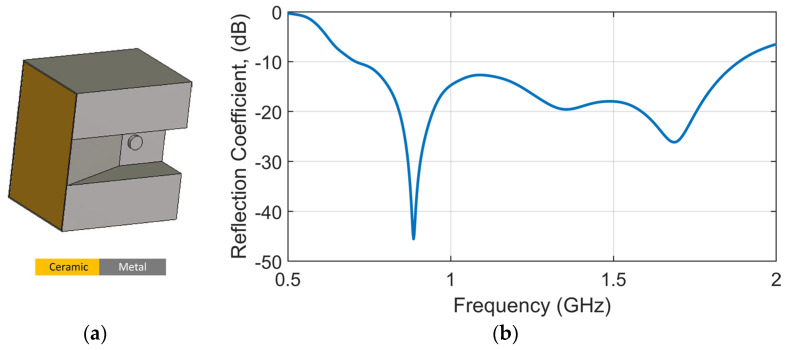
(**a**) Antenna geometry of loaded ceramic waveguide antenna and (**b**) the corresponding simulated reflection coefficient.

**Figure 3:**
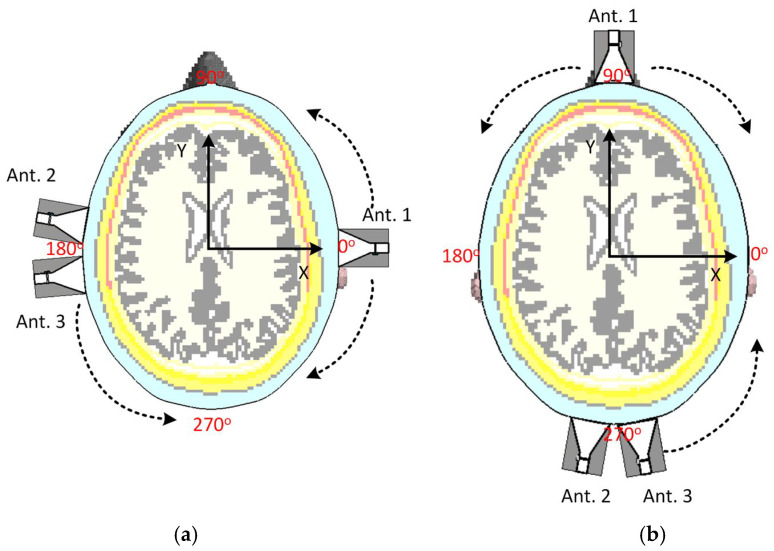
Two different scenarios to test the position of antennas: (**a**) antennas aligned on horizontal axes and (**b**) antennas aligned to the vertical axes. The dotted arrows show the direction of movement of the antennas.

Without losing the generality of the analysis, the antenna used in this study is a ceramic-loaded waveguide antenna [28], specifically redesigned to operate across a wide bandwidth from 0.7 GHz to 1.9 GHz. This frequency range in the lower microwave spectrum was selected to achieve an optimal balance between penetration depth and imaging resolution. The antenna configuration is illustrated in Figure 2a, and its corresponding reflection coefficient is shown in Figure 2b. The structure is fully metalized to suppress back radiation and electromagnetic leakage, thereby ensuring efficient forward radiation and minimizing coupling within the imaging array. To reduce the physical size of the antenna and allow for the integration of a greater number of elements around the human head, a high-permittivity ceramic material (dielectric constant = 50) is embedded within the waveguide cavity, as shown in Figure 3a. This ceramic loading effectively shortens the guided wavelength, enabling compactness without compromising electromagnetic performance. The antenna’s length, width, and height are 36.6 mm, 25.6 mm, and 33.8 mm, respectively.

To further enhance the electromagnetic penetration into the head and maintain efficient power transfer at different positions of the head, a low-loss matching medium is applied between the antenna aperture and the head model. This ensures broadband impedance matching, which is critical for reliable imaging performance. The medium is chosen to approximate the average dielectric properties of the head tissues, with an average relative permittivity of 40 and conductivity of 0.15 S/m at 1 GHz.

### 2.2. Impact of Antenna Positioning on MIC

**Figure 4:**
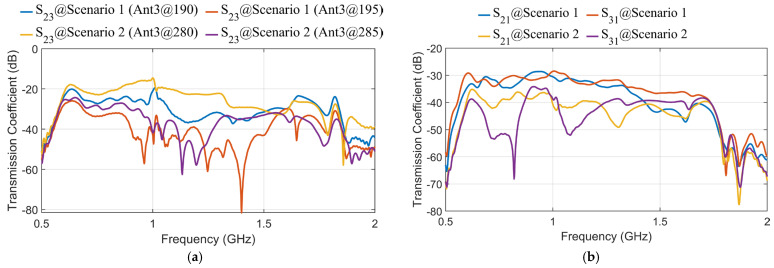
Samples of transmission coefficients in different scenarios: (**a**) S_23_ at different positions of Antenna 3, (**b**) S_21_ and S_31._

In order to initially assess the impact of antenna positions on the acquired data and their independence, two different test scenarios are considered, as shown in Figure 3. A head from MRI Duke voxel-based model with 1 mm×1 mm×1 mm resolution [29] is used in this paper as the domain under test. Three antennas are employed to test the MIC when the antennas are moved slightly. Given that the head has two major axes, two different setup scenarios are designed to evaluate the effect of antenna movement on data capture (see Figure 3). Figure 4 shows how the transmission coefficients between antennas can change significantly due to their relative positions.

In the first scenario, the antennas are positioned around the head model with Antenna 1 (as a transmitter) on the right lateral side of the head at an angle of $0^{o}$, while Antenna 2 and Antenna 3 (as receivers) are positioned on the left lateral side at $180^{o}$. This setup evaluates side-to-side (lateral) signal propagation across the head. In the second scenario, the antennas are rearranged to evaluate front-to-back transmission as shown in Figure 3b. Antenna 1 is located on the anterior side (forehead), and Antennas 2 and 3 are positioned on the posterior side (back of the head). Although the relative distance between transmitting and receiving antennas remains similar, this setup evaluates transmission along a different anatomical axis. In other words, these scenarios aim to evaluate how lateral and vertical movements of the antennas affect the captured data and the independence of the signals received by each antenna. By evaluating the mutual information coefficient (MIC) values in different configurations, we can identify the optimal positions for the antennas to maximize the capture of independent and useful information. This is crucial for improving the overall performance of the MMI system. Specifically, the MIC describes the relationship between different antennas, that is why it is called mutual, meaning $S_{ii}$ is not directly involved in the calculation. Instead, transmission coefficients $S_{ij}$ are used to assess similarity between signals received at different antennas when a specific antenna operates as the transmitter and, thus, estimate the total available information. On the other hand, since $S_{ii}$ usually has a much larger value than $S_{ij}$ due to any mismatch between the antenna and the object and antenna self-reflections, including $S_{ii}$ in MIC calculation can lead to inaccurate results. However, $S_{ii}$ is still considered indirectly; if one antenna has poor matching with the imaged object, it adversely impacts the system’s overall available information and performance. Thus, before any calculation, we ensure an acceptable $S_{ii}$, such as below −10 dB. Thus, MIC is calculated as follows [6]:

(1) $$MIC\left(i,j\right)=\frac{\left|\sum_{f}\;S_{i}\left(f\right)\cdot S_{j}^{*}\left(f\right)\right|}{\sqrt{\sum_{f}\;{\left|S_{i}\left(f\right)\right|}^{2}\cdot \sum_{f}\;{\left|S_{j}\left(f\right)\right|}^{2}}}$$

where i and j refer to the index of the antennas (i ≠ j), while S(f) refers to the captured signals at the antenna at each frequency point f. Table 1 presents the MIC values between Antenna 2 and Antenna 3 for the two different scenarios, highlighting the impact of antenna positioning on information redundancy. Scenario 1 features closely spaced antennas resulting in high MIC values (e.g., 0.8395 at 190°, 0.7134 at 195°), indicating significant data redundancy. In contrast, Scenario 2 (right) exhibits lower MIC values (e.g., 0.112 at 280°, 0.231 at 285°), implying better spatial diversity in the captured data. However, an important limitation of this analysis is that it consistently compares the data from Antenna 3 (as it moves) to a fixed Antenna 2. Since Antenna 2 occupies a unique position and typically faces different tissue compositions than other antenna placements, especially in Scenario 2, the resulting MIC values remain low regardless of where Antenna 3 moves. To make this approach more practical and informative, MIC should not only be evaluated between Antenna 3 and Antenna 2, but also between consecutive positions of Antenna 3 itself. This would help quantify how much new, independent information each new candidate position of Antenna 3 brings relative to its previous location. For example, if Antenna 3 is positioned at 280° with MIC=0.112 when compared to Antenna 2, and then moved to 285°, the MIC compared to Antenna 2 may still be low, but the MIC relative to 280° might be high. So, MIC between consecutive positions can be calculated as follows:

(2) $${MIC}_{n}=MIC\left(\mathrm{Data}\;\mathrm{at}\;\mathrm{Position}\;n,\;\mathrm{Data}\;\mathrm{at}\;\mathrm{Position}\;n-1\right)$$

**Figure 5:**
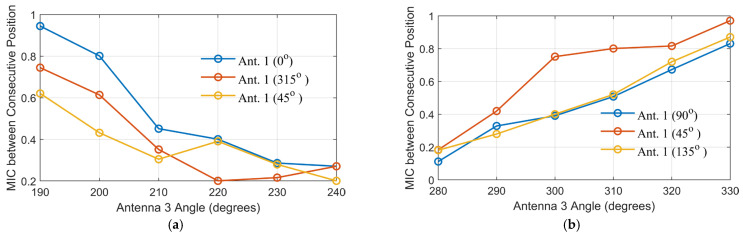
MIC between consecutive positions of Antenna 3 for different angles of Antenna 1 in the two scenarios: (**a**) for Scenario 1 and (**b**) for Scenario 2.

Figure 5 shows the *MIC* between consecutive positions of Antenna 3 as it moves along its angular path in both scenarios, with Antenna 1 fixed at various locations. This analysis evaluates the amount of new, independent information contributed by each incremental movement of Antenna 3, offering insight into the spatial diversity achieved by the scanning configuration. In Scenario 1 (Figure 5a), a simplified scanning scenario was introduced, where Antenna 3 scans from 270° to 330° in 10° increments, while Antenna 1 is placed at 45°, 90°, and 135°. The selected range and step size were chosen to maintain simplicity while being sufficient to highlight the impact of antenna positional changes on the captured information. The MIC values between consecutive positions show relatively high levels, particularly when Antenna 1 is positioned at 135°, suggesting that the information captured during the scanning process is moderately redundant.

On the other hand, Scenario 2 (Figure 5b) demonstrates a more favorable information profile. Antenna 3 scans from 180° to 240°, with Antenna 1 positioned at 0°, 315°, and 45°. The MIC values between consecutive positions are consistently lower than in Scenario 1, indicating greater spatial diversity and less data redundancy. Notably, more independent information is captured when the scanning positions of Antenna 3 are closer to the vertical axis of the head model, particularly evident with certain Antenna 1 positions. This can be attributed to the complex tissue variations and asymmetries encountered near the vertical midline, which naturally introduce more variability in the received signals.

Although the relative positioning of Antennas 1, 2, and 3 remains similar between the two scenarios, the differences in the calculated MIC are primarily driven by the propagation environment, as is clear in the transmission coefficients in Figure 4. In Scenario 1, the electromagnetic waves from Antenna 1 to Antennas 2 and 3 propagate through similar tissue regions. This is clear in S_21_ and S_31_ in Figure 4b. Consequently, even when Antenna 3 changes its angle, the received signals remain highly correlated, resulting in higher MIC values. In contrast, Scenario 2 exhibits greater heterogeneity in the tissues along the propagation paths. Therefore, any small shift in Antenna 3’s position leads to significant changes in the tissue properties encountered, causing variations in the S-parameters and resulting in lower MIC values (see S_21_ and S_31_ in Figure 4b). Therefore, the observed differences in MIC are attributed to the varying degrees of tissue heterogeneity encountered along the wave paths, not solely to the antennas’ geometrical arrangement.

Based on the above-mentioned investigations, a more robust and practical optimization strategy would involve sequentially selecting antenna positions based on low MIC relative to the previously selected antenna, rather than to a fixed reference antenna, to ensure maximal information gain and minimal redundancy across the array. In real scenarios, particularly for multistatic systems where each antenna receives data from all other antennas, this kind of manual analysis becomes impractical. Therefore, developing an automated, data-driven optimization framework that considers these interdependencies will be critical for scalable and effective system design.

## 3. Optimization of Antenna Positions for the Whole System

To achieve an optimal non-uniform antenna array configuration, particle swarm optimization (PSO) is used [30]. PSO is a population-based stochastic optimization algorithm inspired by the social behavior of birds and fish. The objective is to determine the best number of antenna elements and their spatial distribution to enhance imaging performance, where the optimization problem is defined to maximize the total independent information. In MWI systems, each antenna operates as both transmitter and receiver (multistatic mode), and the mutual information between each pair of received signals is computed. Therefore, the goal of the optimization is to minimize the average MIC across all antenna pairs.

(3) Ecost=N2∑i=1N ∑j=i+1N MICi,j

where N  is the number of antennas, and (i,j)  represent the index of the antenna. The cost function ($E_{cost}$) represents the average MIC across all unique antenna pairs. Minimizing cost function results in capturing high independent information across the array.

The PSO was used and managed by the Chaotic inertia weight factors [31]. The swarm evolves iteratively by updating each particle’s position and velocity based on individual and collective experiences. We are starting by initializing a population of P particles with random antenna positions restricted to the domain under test, which is the human head in this study, as follows:

(4) $$X_{k}=\left[x_{1},y_{1},x_{2},y_{2},\ldots ,x_{N},y_{N}\right]$$

The velocity and position of each particle are updated using the following equations:

(5) $$V_{k}\left(t+1\right)=\omega V_{k}\left(t\right)+c_{1}r_{1}\left(P_{k}-X_{k}\left(t\right)\right)+c_{2}r_{2}\left(G-X_{k}\left(t\right)\right)$$

(6) $$X_{k}\left(t+1\right)=X_{k}\left(t\right)+V_{k}\left(t+1\right)$$

where $V_{k}(t)$ is the velocity vector of particle k at iteration t, while w refers to the inertia weight, and $c_{1}$ and $c_{2}$ represent the acceleration coefficients (cognitive and social coefficients)$.\;r_{1}$ and $r_{2}$ are random values in the range [0,1], $P_{k}$ is the best-known position of particle k, and G is the global best position among all particles. $w_{1}$ and $w_{2}$ are the original and final values of inertia weight, respectively. To apply PSO, the acceleration constants are set to $c_{1}=c_{2}=2$, number of variables = 2, maximum iterations T = 500, population size ***P*** = 50. The objective of the PSO algorithm has been expressed as follows:

(7) $$\begin{array}{c} \underset{X}{min}\;E_{\mathrm{cost}}(X)=\frac{2}{N\left(N-1\right)}\sum_{i=1}^{N}\;\sum_{j=i+1}^{N}\;MIC(i,j)\\ \;\mathrm{subject}\;\mathrm{to}\;X_{k}\in \mathrm{antenna}\;\mathrm{region},\;and\;\left\|r_{i}-r_{j}\right\|\geq d_{min},\forall i\neq j \end{array}$$

where $r_{i}$ and $r_{j}$ refer to the position vector of Antenna i and j, respectively. $d_{min}$ is the minimum allowable distance between two antennas.

**Figure 6:**
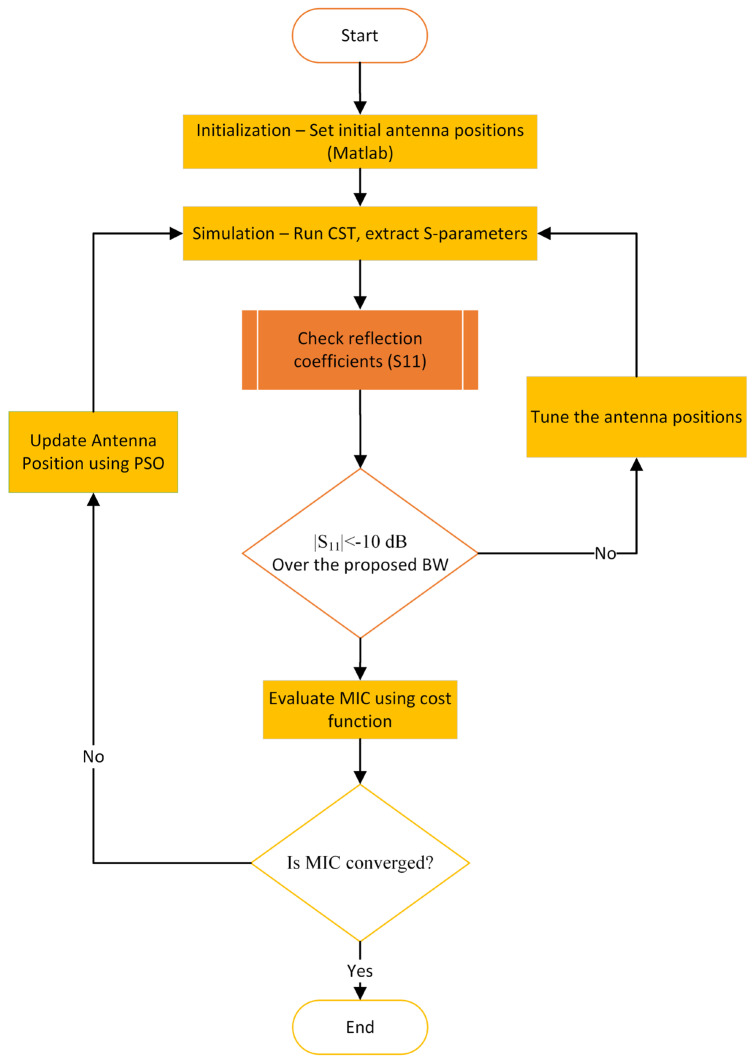
Framework of the proposed imaging antenna array position optimization.

To implement the above-mentioned procedures, the optimization workflow proceeds as follows (see Figure 6):

1. Initialization: begin with an initial set of uniformly distributed antenna positions around the imaging domain.
2. Simulation: Each antenna configuration is simulated using CST. All S-parameters, including both reflection and transmission coefficients, are extracted and transferred to a MATLAB-2024 code for further analysis.
3. Evaluation: In MATLAB, the reflection coefficients (S11) are checked to ensure each antenna maintains the required matching bandwidth (e.g., <−10 dB). The transmission coefficients are then evaluated using a custom-defined cost function (Equation (7)), which quantifies the spatial information diversity captured by the configuration based on the minimum MIC.
4. Optimization: Based on the output of the cost function, the PSO algorithm updates the antenna positions to suggest an improved configuration. This updated layout is fed back into CST for re-simulation.
5. Iteration: Steps 2 to 4 are repeated until the convergence criteria are met, indicating that a near-optimal or satisfactory antenna arrangement has been achieved.

**Figure 7:**
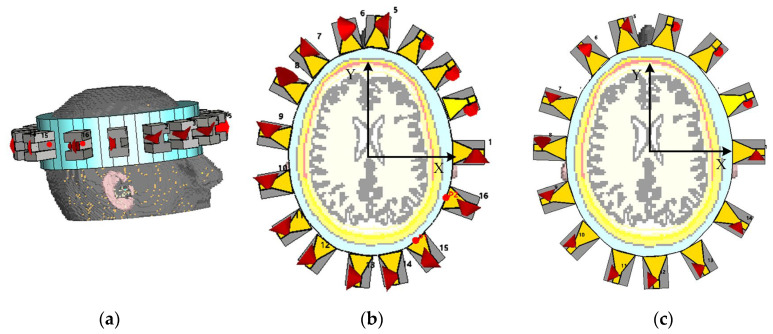
Simulation environment representing a head model with the antenna array systems: (**a**) 3D structure for non-uniform 16 antennas, (**b**,**c**) are xy-cutting views at z = 0 for non-uniform 16 antennas and non-uniform 14 antennas, respectively.

**Figure 8:**
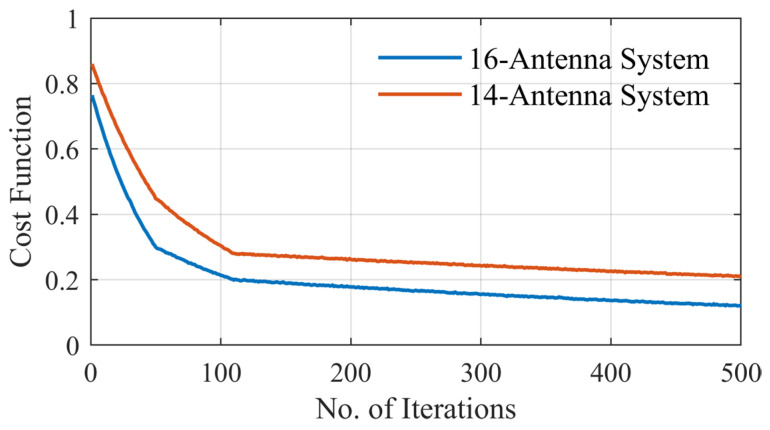
Cost function for non-uniform distributions for the 16-antenna system and the 14-antenna system.

**Figure 9:**
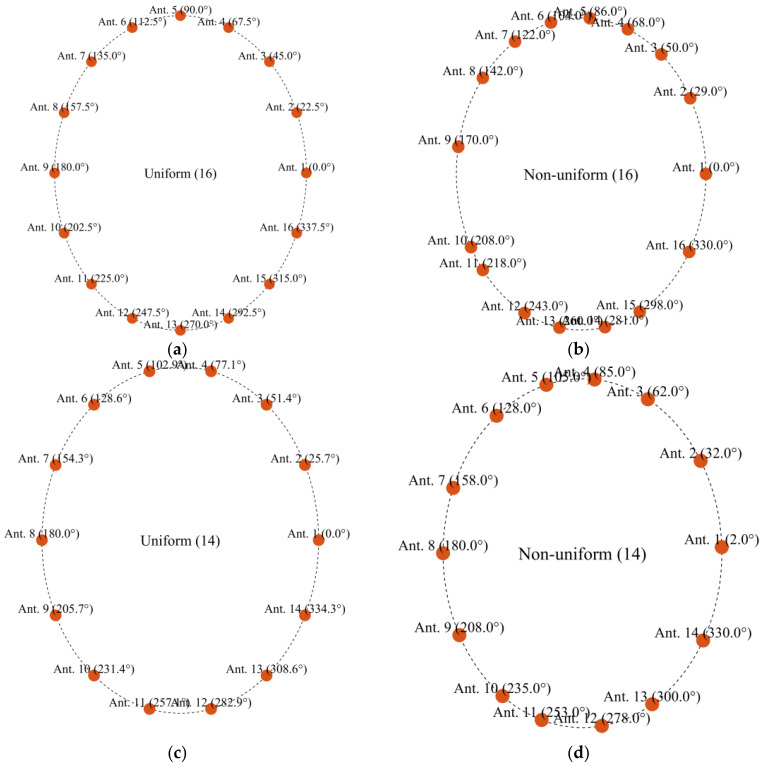
Angular positions of antennas for two different systems for uniform and non-uniform distribution: (**a**) uniform 16, (**b**) non-uniform 16, (**c**) uniform 14, and (**d**) non-uniform 14.

To evaluate the effectiveness of non-uniform antenna configurations in electromagnetic microwave imaging systems, two head imaging setup systems are simulated using CST Microwave Studio (see Figure 7). The first system comprises 16 antennas, whereas the second consists of 14 antennas. Each system is analyzed under a non-uniform configuration, where antenna positions are optimized to capture the maximum amount of unique spatial information based on the above-mentioned optimization approach. Also, the two systems have been analyzed under a uniform configuration for the comparison. The antenna positions in the non-uniform case are derived using the PSO algorithm that minimizes a custom cost function designed to enhance spatial diversity and minimize redundancy between sensing nodes. Figure 8 shows the convergence behavior of the cost function for both the 16-antenna and 14-antenna systems. For the 16-antenna setup, the cost function shows a steep drop from an initial value of approximately 0.78 to below 0.2 within the first 110 iterations, indicating rapid optimization. The curve continues to converge gradually to a final cost of 0.14 after 500 iterations. In comparison, the 14-antenna system follows a similar trend but stabilizes at a slightly higher final cost value of approximately 0.22, reflecting the reduced spatial degrees of freedom due to fewer antenna elements. Compared with the average MIC values for the uniform setups, the MIC value for the uniform 16-antenna configuration is 0.45. This reinforces the need for optimized (non-uniform) placement to better exploit spatial diversity, especially in systems with fewer antennas. Table 2 and Figure 9 show the antenna positions for both uniform and non-uniform systems. It is worth noting that while the optimization procedure illustrated in Figure 6 can be used for any imaged object and antenna array, the optimized positions of the antennas depend on the shape of the imaged object and its anatomy.

## 4. Performance Evaluation and Discussion

**Figure 10:**
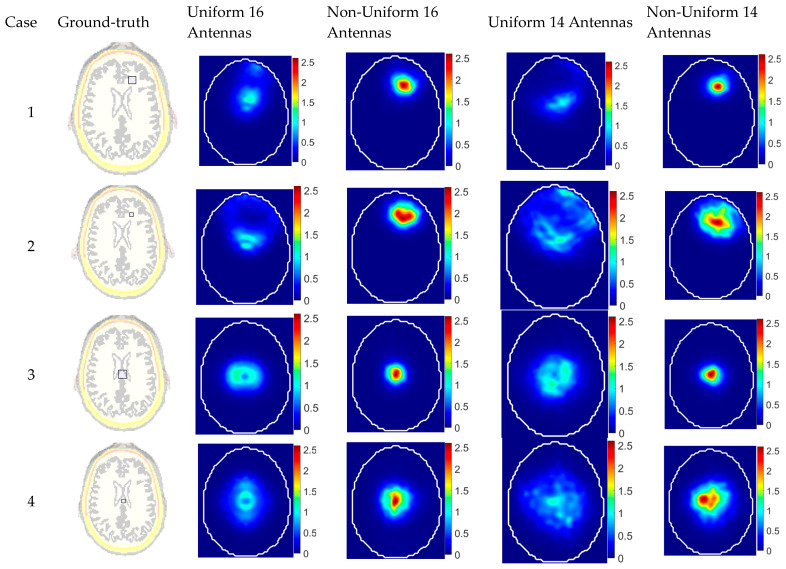
Reconstructed images for different cases using different system configurations compared with the ground truth.

This section presents an evaluation of the different systems: uniform and non-uniform configurations of antenna arrays in detecting abnormalities within a realistic head model. To this end, four abnormal scenarios are tested for each configuration, simulating stroke-like dielectric inclusions of varying sizes and positions. These scenarios represent clinically relevant challenges, including small lesions and strokes. The dielectric properties of the target are chosen to be water, emulating a physiological abnormality, such as a tumor or lesion, within the imaged object. The characteristics of these test cases are summarized in Table 3 and illustrated in Figure 10. The targets were located either centrally (at (0, 0) mm) or peripherally (at (15, 50) mm) and varied in size between small ($5\times 5\times 40\;mm^{3}$) and large ($10\times 10\times 40\;mm^{3})$.

For image reconstruction, a confocal imaging algorithm is used. This algorithm discretizes the imaging domain into m pixels and focuses the received time–domain signals based on the estimated signal propagation delays from each antenna to each pixel. The delay time $\tau_{q}$ from the $q^{th}$ antenna to the $m^{th}$ pixel is computed as follows:

(8) $$\tau_{q}=\frac{\left\|a_{q}-i_{m}\right\|.\sqrt{\epsilon_{eff}^{q}}}{c}$$

where $a_{q}$ and $i_{m}$ denote the spatial coordinates of the antenna and pixel, respectively, $\epsilon_{eff}^{q}$ is the effective permittivity of the imaging domain for the $q^{th}$ antenna, and c is the speed of light in a vacuum. The effective permittivity is selected using the optimization methodology introduced by the authors in [25]. Due to the inherent signal attenuation in lossy biological tissues, the adaptive version of the multistatic confocal imaging algorithm includes a weighting factor that is employed to adjust the signal contributions based on the distance between each antenna and each pixel [32]. The imaging algorithm processes the full S-parameter matrix, including both reflection coefficients ($S_{ii}$) and transmission coefficients ($S_{ij}$), ensuring that all available spatial and spectral information contributes to the final image reconstruction. The image intensity at each pixel is computed using the following equation:

(9) $$I\left(i_{m}\right)=\sum_{q=1}^{Q}\;\sum_{w=-W}^{W}\;\left|\left\|a_{q}-i_{m}\right\|\cdot t_{q}\left(2\tau_{q}+t_{in}+w\right)\right|$$

where Q is the total number of antennas, W is the summation window length, and $t_{in}$ is an empirically determined internal delay that accounts for the physical offset between the antenna’s aperture and the receiver port. In the reconstructed images, $t_{in}=1\;ns$ and W=30 are selected to achieve optimum image quality. The reconstructed images are generated using differential signals between healthy and unhealthy heads to remove clutter.

To evaluate the accuracy of the reconstructed images, two quantitative metrics are used [33]: (1) signal-to-clutter ratio (SCR), which is the ratio between the maximum intensity at the detected target area in the unhealthy domain to the maximum intensity of the corresponding area in the healthy domain and (2) position error (PE), which is the absolute difference between the centers of the actual and detected target. The two factors are defined as follows:

(10) SCR=10log10⁡max⁡Iimmax⁡Inin∀im∈D∀in∈Dn

(11) $$PE=\left|\rho^{*}-\alpha \right|$$

where *I* (.) represents the intensity at each pixel within the imaging domain, while *D* and $D_{n}$ refer to the target (tumor) area in the unhealthy domain and its corresponding location in the area in the healthy domain, respectively. $\rho^{*}$ and α are the centers of the detected and actual target region, respectively. For ideal detection, the criteria are SCR≫0 dB and PE=0 mm.

The imaging results clearly demonstrate the superior performance of the non-uniform antenna configurations in both shallow and central target detection scenarios (see Figure 10). In Cases 1 and 2, the non-uniform 16-antenna system achieved significantly better localization and image clarity, with a perfect position error (PE = 0 mm) and the highest signal-to-clutter ratio (SCR = 6.45 dB) in Case 1, while the uniform counterpart mislocated the target by 28.6 mm despite a comparable SCR. This trend was even clearer in Case 2, where the uniform system failed to accurately localize the small target (PE = 48.1 mm) and produced a very low SCR (0.51 dB), whereas the non-uniform system maintained a low PE (2 mm) and a good SCR (4.56 dB). For central targets (Cases 3 and 4), both configurations show improved accuracy, but the non-uniform setup still outperforms the uniform one, achieving minimal PE (1 mm) and higher SCRs (6.78 dB and 4.86 dB), compared to the larger errors and lower contrast in the uniform system. These findings highlight the effectiveness of optimizing antenna placement in enhancing microwave imaging accuracy and robustness, especially when dealing with variable target positions and sizes.

One of the significant insights from the reconstructed images is that the proposed approach of optimized non-uniform imaging antenna array enables using a smaller number of antenna elements for building portable devices without sacrificing the imaging quality. A system with 14 antennas instead of 16 antennas, when optimally distributed, can outperform a 16-antenna system with uniform placement. In Case 1, the non-uniform 14-antenna setup achieved a PE of 4 mm and an SCR of 4.15 dB, while the uniform 16-antenna system showed a much higher PE of 28.6 mm and a similar SCR of 6.18 dB. In Case 2, the non-uniform 14-antenna system had a PE of 10 mm and an SCR of 2.81 dB, compared to the uniform 16-antenna system, which exhibited a poor localization (PE = 48.1 mm) and an SCR of only 0.51 dB. These results highlight that the non-uniform 14-antenna configuration outperforms the 16-antenna uniform setup in terms of both localization and clarity, as evidenced by the consistently better PE and SCR values, while the uniform 14-element array failed to localize the target accurately (PE = 43.17 mm, SCR = 0.317 dB). Beyond improved imaging performance, this finding also suggests that a smaller number of antennas can reduce the overall system complexity, cost, and power consumption, making it a more practical and efficient solution for portable, real-world biomedical imaging applications.

It is important to note that the coupling medium and antenna structure presented here serves solely as an illustrative example to demonstrate the feasibility of the proposed concept. This study’s primary focus is on introducing and validating the effectiveness of non-uniform antenna arrays for improving the performance of medical microwave imaging systems. The proposed framework is general and adaptable and can be applied to a variety of anatomical regions (e.g., head, knee, torso, breast) and imaging setups. In each case, the selection of the coupling medium and antenna structure can be tailored to the application to ensure proper impedance matching and wave penetration. Also, the number of antennas is influenced by the size and shape of the imaging region (e.g., head, torso, knee). While using a large number of imaging antennas can increase spatial sampling, it often leads to redundant and correlated data. This study shows that with the same number of antennas or fewer, a non-uniform, optimized spatial distribution significantly improves the independence of the captured signals, as shown by reduced mutual information. This results in better image clarity and localization accuracy compared to uniformly distributed arrays.

## 5. Conclusions

Non-uniform antenna arrays have been proposed to enhance the accuracy of medical microwave imaging. By selecting the optimal position of each element in the antenna array, the spatial diversity can be enhanced while information redundancy is highly reduced, leading to improved imaging performance. The mutual information coefficient (MIC) is used to evaluate and minimize redundancy between received signals, ensuring the capture of independent and useful information. PSO is implemented to determine the optimal number and positions of antennas, achieving minimal MIC values across the array. The reconstructed images for different scenarios demonstrate that non-uniform antenna configurations significantly outperform uniform setups in terms of localization accuracy and signal-to-clutter ratio. Notably, a 14-antenna non-uniform system demonstrates superior performance compared to a 16-antenna uniform system, highlighting the efficiency and practicality of optimized placement. This approach not only improves diagnostic accuracy but also reduces system complexity, cost, and power consumption, making it a viable solution for real-world biomedical imaging applications.

## Figures and Tables

**Table 1:** MIC values between Antenna 2 and Antenna 3 in different scenarios.

Antenna 3 @ Scenario 1	MIC (2,3)	Antenna 3 @ Scenario 2	MIC (2,3)
$190^{{}^{\circ}}$	0.840	$280^{{}^{\circ}}$	0.112
$195^{{}^{\circ}}$	0.713	$285^{{}^{\circ}}$	0.231
$200^{{}^{\circ}}$	0.709	$290^{{}^{\circ}}$	0.291
$205^{{}^{\circ}}$	0.512	$295^{{}^{\circ}}$	0.395
$210^{{}^{\circ}}$	0.387	$300^{{}^{\circ}}$	0.413
$215^{{}^{\circ}}$	0.781	$305^{{}^{\circ}}$	0.231
$220^{{}^{\circ}}$	0.660	$310^{{}^{\circ}}$	0.201
$225^{{}^{\circ}}$	0.321	$315^{{}^{\circ}}$	0.321

**Table 2:** Angular positions of antennas for two different systems for uniform and non-uniform distribution.

	Uniform (16)	Non-Uniform (16)	Uniform (14)	Non-Uniform (14)
1	$0^{{}^{\circ}}$	$4^{{}^{\circ}}$	$0^{{}^{\circ}}$	$2^{{}^{\circ}}$
2	$22.5^{{}^{\circ}}$	$29^{{}^{\circ}}$	$25.7^{{}^{\circ}}$	$32^{{}^{\circ}}$
3	$45^{{}^{\circ}}$	$50^{{}^{\circ}}$	$51.4^{{}^{\circ}}$	$62^{{}^{\circ}}$
4	$67.5^{{}^{\circ}}$	$68^{{}^{\circ}}$	$77.14^{{}^{\circ}}$	$85^{{}^{\circ}}$
5	$90^{{}^{\circ}}$	$86^{{}^{\circ}}$	$102.85^{{}^{\circ}}$	$105^{{}^{\circ}}$
6	$112.5^{{}^{\circ}}$	$104^{{}^{\circ}}$	$128.5^{{}^{\circ}}$	$128^{{}^{\circ}}$
7	$135^{{}^{\circ}}$	$122^{{}^{\circ}}$	$154.28^{{}^{\circ}}$	$158^{{}^{\circ}}$
8	$157.5^{{}^{\circ}}$	$142^{{}^{\circ}}$	$180^{{}^{\circ}}$	$183^{{}^{\circ}}$
9	$180^{{}^{\circ}}$	$170^{{}^{\circ}}$	$205.7^{{}^{\circ}}$	$208^{{}^{\circ}}$
10	$202.5^{{}^{\circ}}$	$200^{{}^{\circ}}$	$231.4^{{}^{\circ}}$	$235^{{}^{\circ}}$
11	$225^{{}^{\circ}}$	$218^{{}^{\circ}}$	$257.14^{{}^{\circ}}$	$253^{{}^{\circ}}$
12	$247.5^{{}^{\circ}}$	$243^{{}^{\circ}}$	$282.85^{{}^{\circ}}$	$278^{{}^{\circ}}$
13	$270^{{}^{\circ}}$	$263^{{}^{\circ}}$	$308.5^{{}^{\circ}}$	$300^{{}^{\circ}}$
14	$292.5^{{}^{\circ}}$	$281^{{}^{\circ}}$	$334.28^{{}^{\circ}}$	$330^{{}^{\circ}}$
15	$315^{{}^{\circ}}$	$298^{{}^{\circ}}$		
16	$337.5^{{}^{\circ}}$	$330^{{}^{\circ}}$		

**Table 3:** Evaluation of the reconstructed images for different cases from different systems.

Case	Target Position (x,y)/Target Size *w* × *l* × *h* (mm)	Uniform 16 Antennas		Non-Uniform 16 Antennas		Uniform 14 Antennas		Non-Uniform 14 Antennas	
		PE	SCR	PE	SCR	PE	SCR	PE	SCR
1	(15, 50)/(10×10×40)	28.6 mm	6.18 dB	0 mm	6.45 dB	34.05 mm	2.33 dB	2 mm	5.68 dB
2	(15, 50)/(5×5×40)	48.1 mm	0.51 dB	2 mm	4.56 dB	43.17 mm	0.317 dB	4 mm	3.71 dB
3	(0, 0)/(10×10×40)	15 mm	1.81 dB	1 mm	6.78 dB	5.385 mm	1.515 dB	1 mm	5.41 dB
4	(0, 0)/(5×5×40)	12.1 mm	4.14 dB	1 mm	4.86 dB	16.76 mm	0.827 dB	8.7 mm	2.86 dB

## Data Availability

All the data are included in the article.
